# The Mechanisms of the Anti-Inflammatory and Anti-Apoptotic Effects of Omega-3 Polyunsaturated Fatty Acids during Methotrexate-Induced Intestinal Damage in Cell Line and in a Rat Model

**DOI:** 10.3390/nu13030888

**Published:** 2021-03-10

**Authors:** Tal Koppelmann, Yulia Pollak, Yoav Ben-Shahar, Gregory Gorelik, Igor Sukhotnik

**Affiliations:** 1 Laboratory of Intestinal Adaptation and Recovery, Department of Paediatric Surgery, Dana-Dwek Children’s Hospital, Tel Aviv Sourasky Medical Center, 6 Weizmann st, Tel Aviv 6423906, Israel; talkopp@gmail.com (T.K.); yuliapo@tlvmc.gov.il (Y.P.); yoav_bs@hotmail.com (Y.B.-S.); 2 Dept Pathology, Tel Aviv Sourasky Medical Center, Tel Aviv 6423906, Israel; gregoryg@tlvmc.gov.il; 3 Sackler Faculty of Medicine, Tel Aviv University, Ramat Aviv, Tel Aviv 69978, Israel

**Keywords:** chemotherapy, intestine, mucositis, TNF-α, NF-κB, COX-2, apoptosis

## Abstract

*Background:* The aim of this study was to examine the anti-inflammatory and anti-apoptotic patterns of omega-3 polyunsaturated fatty acids (n-3 PUFAs) during methotrexate (MTX) induced intestinal damage in cell culture and in a rat model. *Methods:* Non-treated and treated with MTX HT 29 and HCT116cells were exposed to increasing doses of n-3 PUFAs and cell viability was evaluated using PrestoBlue^®^ assay. Male Sprague-Dawley rats were divided into 4 experimental groups: Control rats, CONTR+n-3 PUFA rats that were treated with oral n-3 PUFA, MTX rats were treated with MTX given IP, and MTX+n-3 PUFA rats were treated with oral n-3 PUFA before and following injection of MTX. Intestinal mucosal parameters and mucosal inflammation, enterocyte proliferation and apoptosis, TNF-α in mucosal tissue and plasma (ELISA), NF-κB, COX-2, TNF-α, Fas, FasL, Fadd, Bid, Bax and Bcl-2gene and protein levels were determined 72 h following MTX injection. *Results:* Exposure of HT 29 and HCT116cells to n-3 PUFA attenuated inhibiting effects of MTX on cell viability. MTX-n-3 PUFA rats demonstrated a lower intestinal injury score and enhanced intestinal repair. A significant decrease in enterocyte apoptosis in MTX+n-3 PUFA rats was accompanied by decreased TNF-α, FAS, FasL, FADD and BID mRNA levels. Decreased NF-κB, COX-2 and TNF-α levels in mucosa was accompanied by a decreased number of IELs and macrophages. *Conclusions:* n-3 PUFAs inhibit NF-κB/COX-2 induced production of pro-inflammatory cytokines and inhibit cell apoptosis mainly by extrinsic pathway in rats with MTX-induced intestinal damage.

## 1. Introduction

Chemotherapy-induced mucositis is a known side effect of cytoreductive cancer therapy. It occurs in 10–40% of patients, rising to about 80–90% for patients with head and neck cancer treated with both radio- and/or chemotherapy [1,2]. Mucositis often leads to delay or discontinuation of selected antineoplastic treatment, reduces chemotherapy regimen decreasing patients’ chances of survival. It also impairs nutritional status leading to weight loss. All these may result in lengthening of hospital stay and increase in healthcare costs leading to impairment of life quality, and even fatality. Recent years have shed light on underlying mechanisms causing mucositis, but there are still many to be revealed. Since the intestinal epithelium is a highly proliferative tissue and continuously renews every 4–5 days, proliferative cells (intestinal stem cells, ISCs) may be targeted by chemotherapy the same way it targets DNA double-strand break and repair pathways in tumor cells [3]. Chemotherapy may also cause cell damage through production of pro-inflammatory cytokines, chemokines and angiogenic factors such as TNF-α, IL-1β and IL-6, or through generation of reactive oxygen species [4]. Cumulatively, these pro-inflammatory activities lead to tissue injury and cell apoptosis [4]. Apoptosis is a tightly regulated process of programmed cells death, which is characterized by membrane blebbing, cell shrinkage and nuclear disintegration, leading to formation of apoptotic bodies, which can be engulfed by immune cells. Apoptosis can follow an intrinsic pathway or an extrinsic pathway each triggered by specific stimuli. [5]. The intrinsic pathway, also known as the mitochondrial pathway, is related to the mitochondrial respiratory chain and the mitochondrial metabolism. The intrinsic pathway is initiated by a stimuli to the internal mitochondria membrane that provides energy for the whole process. This pathway is regulated by several genes (Bax-Bcl-2 family) some active as pro-apoptotic, others as anti-apoptotic genes. Recent research has provided evidence to the important role that Bcl-2 plays in the regulation of programmed cell death during chemotherapy induced mucositis [6]. The extrinsic pathway of apoptosis is triggered by the oligomerization of transmembrane proteins derived from the family of death receptors which are a part of the tumor necrosis factor (TNF) receptor superfamily [7]. The most well-known members of this superfamily are TNF-α, Fas, FasL, TNFR1, DR3-6. In the normal gastrointestinal tract, TNF-α acts as a mediator for cell survival by the activation of nuclear factor-κB (NF-κB signaling). On the other hand, TNF-α might trigger programmed cell death through both caspase-dependent and caspase-independent pathway. By regulating these cell survival and cell death mechanisms, TNF-α exerts a variety of beneficial functions in the intestine. Several studies have previously shown that epithelial cell apoptosis in the setting of intestinal mucositis is driven mainly by TNF-α superfamily [8]. The released TNF-α activates MAPK (mitogen activated protein kinase) on target cells and sustains NF-κB activity resulting in cell death (pro-apoptotic effects) and further damage to mucosa and sub mucosa structures.

Polyunsaturated fatty acids (PUFA) has emerged as a subject of scientific research and public interest over the past three decades. These biologically essential macromolecules are involved in many physiologic and metabolic processes. Omega-3 FAs are characterized by their specific chemical structure that consists of a double bond in at three-carbon atom away from the terminal (the omega) methyl group and consist of long-chain and short-chain FAs [9]. such as docosahexaenoic acid (DHA; 22:6 n-3) and eicosapentaenoic acid (EPA; 20:5 n-3) are two examples of long-chain n-3 PUFAs that are mainly found in fatty fish such as salmon, tuna, mackerel, halibut, herring, and may be collectively referred to as marine n-3 PUFA. Examining the inflammatory processes, we direct our focus on a precursor of inflammatory eicosanoids (prostaglandin E2 and leukotriene B4) such is the arachidonic acid (AA). We also examine the n-3 PUFAs (EPA and DHA) that inhibits AA metabolism of inflammatory eicosanoids. n-3 PUFAs encourage anti-inflammatory mediators, exert positive effect on leukocyte chemotaxis and inflammatory cytokine production and alter the activity of transcription factors [10]. Fish oil has previously demonstrated the ability to to diminish colonic damage and inflammatory process in animal models of colitis [11] and in patients with inflammatory bowel disease [12] by mediating changes into inflammatory processes in the mucosal cell of intestines. Recent evidence suggests that n-3 PUFAs may act in addition to chemo and/or radiotherapy to kill tumor cells by enhancing the cytotoxicity of chemotherapy drugs, by increasing oxidative stress, or by reducing angiogenesis, inflammation, and metastasis induction [13].

We have recently demonstrated that treating rats with omega-3 pre and post injecting methotrexate (MTX) which is known to cause intestinal damage resulted in less intestinal injury and in improved mucosal recovery [14]. Accelerated cell proliferation and inhibited cell apoptosis were responsible for this positive effect. The purpose of this study, which is a direct continuation of our previous experiment was to investigate the mechanisms of anti-inflammatory and anti-apoptotic effects of n-3 PUFA during MTX-induced intestinal mucositis in a rat model and in a cell culture. 

## 2. Materials and Methods

### 2.1. Reagents

Eicosapentaenoic acid (EPA, #90110) and cervonic acid (DHA, #90310) were purchased from Cayman Chemical (Ann Arbor, MI, USA), dissolved in ethanol and stored at −20 °C. Methotrexate hydrate MTX (M8407) was purchased from Sigma–Aldrich (Rehovot, Israel), dissolved in PBS and stored at −20 °C.

### 2.2. Cell Cultures 

Two kinds of intestinal epithelial cells, HT 29 (ATCC^®^ HTB-38, human) and HCT 116 (ATCC^®^ CCL-247™, human) were purchased from ATCC (Biological Industries Israel Beit Haemek Ltd., Beit Haemek, Israel). Both kinds of cells were cultured in Dulbecco’s modified Eagle’s medium and were supplemented with 10% heat-inactivated fetal bovine serum (Invitrogen, Basel, Switzerland). Cells were kept at a humidified atmosphere with 5% CO_2_ at 37 °C. Containing Medium was changed twice a week in order to maintain an exponential growth phase. The cells were used no more than 10 passages in order to maintain their biological characteristics

### 2.3. Cell Viability and Treatment

The effects of MTX and n-3 PUFA (DHA and EPA) on cell viability were evaluated using PrestoBlue^®^ assay (Molecular Probes, Invitrogen, Eugene, OR, USA). Absorbance of resorufin (converted from resazurin dye) was measured using a BioTek ELx800 Absorbance Microplate Reader (BioTek Instruments, Inc., Winooski, VT, USA) at 570 nm and normalized to 600 nm values. This method allows one to acurately estimate the number of metabolically active cells based upon the absorption of the dye. The following equation was used to calculate cell viability in respect to compound concentration based on absorbance readings: Cell viability (%) = [Absorbance of Treated Sample − Absorbance of Blank] × 100/[Absorbance of Negative Control − Absorbance of Blank].

**Figure 1:**
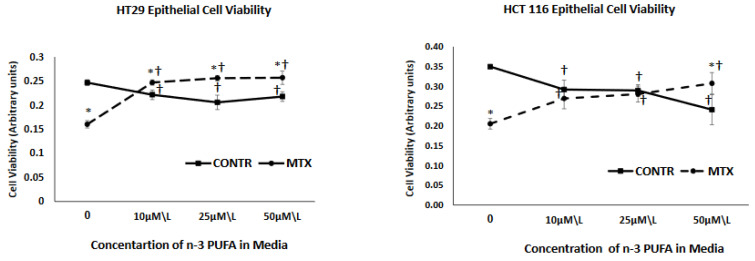
The effect of MTX and increasing concentrations of n-3 PUFAs on HT 29 and HCT 116 cell viability. PrestoBlue^®^ assay was used to evaluate cell viability. MTX-methotrexate, n-3 polyunsaturated fatty acids. Values are mean ± SEM. * *p* < 0.05 MTX-treated vs. MTX-non-treated cells, ^†^ *p* < 0.05 viability late vs. initial.

We conducted our Viability test of cell lines with supplements of separate DHA, EPA and DHA/EPA (a mix in ratio of 3:2) at concentrations of 10 µmol/L 25 µmol/L and 50 µmol/L and found that changes in viability of cells could be noted in concentrations as low as 10 µmol/L (seen in Figure 1). Based on that, and on previous studies [15,16], we used EPA, DHA and DHA\EPA (3:2) at 10, 25 or 50 μmol/L to treat cells at 72 h before administering MTX (250 nM\L and 500 nM\L) or vehicle exposure and a second treatment at the time of administering MTX.

### 2.4. Animals

This study was approved by the Animal Ethics Committee of Tel Aviv Sourasky Medical Center (Ichilov). All Our experiments meticulously followed the guidelines stated in the “Guide for the Care and Use of Laboratory Animals”. Adult male Sprague-Dawley rats weighing 260–280 g were acclimatized prior to the experiment in a 12-h day-night rhythm at 22 °C and a relative humidity of 40–50% for 3–5 days. 

### 2.5. Experimental Design

The experiment was built of four animal groups randomly assigned (8 rats in each) Group A rats (CONTR) were oraly fed canola oil (1 mL) once daily given by orogastric (OG) tube for six days. At day three they received a single 1 mL dose of saline as an intraperitoneal injection. Group B rats (CONTR+n-3 PUFA) were fed once daily with 300 µg/kg/day of n-3 PUFA (EPA 180 µg + DHA 120 µg) by OG tube. At day three they also received a single 1 mL dose of saline as an intraperitoneal injection. Group C rats (MTX) were fed canola oil similar to Group A and received a single intraperitoneal dose of MTX (25 mg/kg) on day three. Group D rats (MTX-n-3 PUFA) were also daily fed with n-3 PUFA (similar to Group B) and IP injected with MTX on day three. The experiment continued for 6 days in total as described and on the seventh day, the animals were euthanized by carbon dioxide (CO_2_) inhalation in accord to AVMA Guidelines for the Euthanasia of Animals. 

### 2.6. Body Weight Gain and Intestinal Mucosal Parameters

We calculated body weight (BW) gain as a BW change [Final BW−Initial BW] and presented it as its percentage of the original BW. Small bowel was excised quickly. Intestinal segments from proximal jejunum and distal ileum were excised for further examination. We weighed Each excised segment and continued to weigh only the mucosa which was scraped carefully off the bowel. Mucosal samples were kept at −70 °C for further investigations. 

### 2.7. Microscopic Intestinal Parameters and Microscopic Assessment of Inflammation

We fixed the proximal jejunum and distal ileum tissue samples in 4% neutral-buffered formalin, embedded in paraffin and sectioned. After deparaffinizing the samples, 5 μm sections were sliced and staining with hematoxylin and eosin was obtained. Each specimen was examined under an optical microscope (10 × 100 magnification). Villus height and crypt depth were measured using an objective mounted micrometer (100× magnification). Mean length was calculated based on 10 separate measurements of villus height and crypt depth. We estimated the damage to intestinal mucosa using intestinal injury score as described by Kesik et al. [17], Evaluating the following parameters: (1) degeneration of surface and crypt epithelium, (2) degeneration of villus structure, and (3) inflammatory cell infiltration of propria. Scores were assigned for each criterion as 0 (none), 1 (mild), 2 (moderate), and 3 (severe) reaching a maximum possible score of 9. Immunohistochemistry with anti-CD 68 antibody (dilution 1:100, ab 125212) was performed in order to color and identify macrophages. We expressed the number of macrophages as the number of positively-stained cells in lamina propria in 10 villi. The number of intraepithelial lymphocytes (IELs) was assessed using H&E staining and was calculated per 10 villi. 

### 2.8. Real-Time PCR

Total RNA from intestinal mucosa was extracted using NucleoZOL (740404, Macherey-Nagel, Düren, Germany), qualified and quantified by spectrometry. We used a qScript cDNA Synthesis Kit (AprexBio, Houston, TX, USA) to reverse transcribe a fixed amount of RNA (1 μg in a total volume of 20 µL) into cDNA. The cDNA was then amplified by a PCR-Thermal Cycler (2720 Thermal Cycler, ABI Applied Bio Systems, Foster City, CA, USA). Quantitative real-time PCR QUANTSTUDIO 1 (Applied Bio Systems, Foster City, CA, USA) was used to determine gene Expression, using SYBR Green Fast Mix (#96074, Quanta Bio, Beverly, MA, USA). Primers for *Rattus norvegicus* TNF-α, FAS, FasL, Fadd, BID, Casp7, GAPDH and Tubulin genes were synthesized by IDT-Integrated-DNA Technologies (Coralville, IA, USA). We used the The ΔΔCt method, normalized to housekeeping genes GAPDH and tubulin, in order to to calculate relative changes in expression of genes.

### 2.9. TNF-α Measurements in Plasma and Intestinal Tissue (Enzyme-Linked Immunosorbent Assay, ELISA)

Levels of TNF-α in plasma and mucosal tissue were determined using an ELISA kit (R&D Systems, Minneapolis, MN, USA). Plasma was centrifuged (20 min at 1000× *g*) and stored at −80 °C for further analyses. Each intestinal mucosal specimen was homogenized in 150 µL Cell Lysis Buffer 2 We incubated samples for1 h on ice. The homogenates were clarified by centrifugation at 4 °C (15,000× *g* for 5 min). We collected 1 µL of the supernatant to determine the total protein concentration using Bio-Rad protein assey (Bio-Rad, Hercules, CA, USA) and the rest of the supernatant fraction we and stored at −80 °C. All steps of the assay in rat TNF-α followed manufacturer’s instructions. 

### 2.10. Enterocyte Proliferation and Apoptosis

We injected 5-bromodeoxyuridine (5-BrdU) standard labeling reagent at a dose of 1 mL/100 g body weight intraperitoneal 2 h prior to sacrifice in order to assess crypt cell proliferation. The intestinal tissue sections (5 μm from proximal jejunum and distal ileum) were stained with a biotinylated monoclonal anti-BrdU antibody using a commercial kit (Zymed Laboratories, Inc, San Francisco, CA, USA). The number of proliferating cells was calculated 10 crypts. Additional 5 μm thick sections were used to investigate apoptotic processes within the enterocytes. We used Caspase-3 as a marker for cell apoptosis, staining our specimens with caspase-3 cleaved concentrated polyclonal antibody (dilution 1:100), and counting the number of apoptotic cells measured in 10 villi. The abovementioned measurements were all done by a pathologist that was blinded to the different experimental rat groups.

### 2.11. Western Blotting 

We used a Bio-Rad Mini-PROTEIN TGX Gels and Trans-Blot Turbo Transfer to perform our immunoblots. The intestinal epithelial cells were isolated from jejunum and ileum. We then added RIPA lysis buffer and a mixture of protease and phosphatase inhibitors, homogenizing them all. Protein concentrations were determined by Bradford reagent (Bio-Rad). Individual samples (30 μg/lane) were loaded on 12% (SDS-PAGE) gels. The membranes were incubated overnight with anti-Bcl-2 antibody (1:1000 dilution, sc-7382), anti-Bax antibody (1:1000 dilution, sc-7480), (Santa Cruz Biotechnologies, Dallas, TX, USA) anti-NF-κB p65 (1:1000; #8242, Cell Signaling, Danver, MA, USA ) and γ-tubulin (1:5000 dilution, T6557 Sigma), (Aldrich, Merk, Darmstadt, Germany). The membranes were then mounted with a secondary antibody, and incubated again for one hour at room temperature (dilution 1:10,000 anti-mouse HRP and anti-rabbit HRP). We then added the t chemiluminescent reagent in order to identify the specific protein bands represented by different optical density, using image analysis software (Image Lab Software version 6.0.1 by Bio-Rad). Calculations of quantity were presented as a ratio to tubulin protein expression.

### 2.12. Statistical Analysis

All data are expressed as mean ± SEM. We used one-way ANOVA for comparison between the four groups, followed by Tukey’s test for pair-wise comparison. Prism software was used (GraphPad Software, Inc., San Diego, CA, USA) and statistical significance was defined as *p* < 0.05.

## 3. Results

### 3.1. Effect of MTX and n-3 PUFA on Intestinal Epithelial Cell Viability

As demonstrated in Figure 1, treatment with MTX resulted in a significant decrease in cell viability for both HT 29 (36% decrease, *p* < 0.001) and HCT 116 (40% decrease, *p* < 0.001) cell lines in comparison to vehicle treated control cells. Exposure of control (non-treated with MTX) cells to three different concentrations of n-3 PUFA resulted in a decreased cell viability that was concentration independent for HT 29 cells (reaching minimal value at a concentration of 25 µm/L but increasing at a concentration of 50 µm/L) and was concentration depended for HCT 166 cells, showing a gradual decrease and reaching a minimal value at a concentration of 50 µm/L. Effect of increasing concentrations of n-3 PUFA on cell viability of MTX-treated cells was different from that of control cells treated with vehicle alone. Exposure of these cells to n-3 PUFA attenuated inhibiting effects of MTX on cell viability. MTX+n-3 PUFA cells showed an increased cell viability that was concentration independent for HT 29 cells (reached maximal value at concentration of 10 µm/L and remained unchanged at both 25 and 50 µm/L concentrations) and was concentration depended for HCT 116 cells, showing gradual increase and reaching maximal value at concentration of 50 µm/L. 

### 3.2. Animal Study

#### 3.2.1. Body Weight Gain and Macroscopic Intestinal Appearance

Similar to our previous experiment [14], Group C-rats that were treated with MTX exhibited a three-fold decrease in body weight gain (*p* = 0.005) compared to control (Group A) (Table 1). Treatment of MTX rats with n-3 PUFA (Group D) led to a significant two-fold increase in body weight gain (*p* < 0.05) compared to Group C—rats that were injected with MTX but did not receive n-3 PUFA. Administration of n-3 PUFA in control animals (Group B) had no significant effect on macroscopic intestinal appearance compared to control rats (Group A) (Table 1). MTX-induced intestinal damage (Group C) led to a significant decrease in jejunal (23%, *p* < 0.05) and ileal (19%, *p* < 0.001) bowel weight as well as a decrease in jejunal (38%, *p* < 0.05), and ileal (27%, *p* < 0.05) mucosal weight in comparison with control animals (Group A) (Table 1). A significant increase in jejunal (30%, *p* < 0.05) and ileal (17%, *p* < 0.05) bowel weight were noted in group D- MTX+n-3 PUFA in comparison to MTX group (C) another significant difference between these groups was notedin jejunal (49%, *p* < 0.05) and ileal (38%, *p* = 0.01) mucosal weights.

#### 3.2.2. Histological Changes

N-3 PUFA administration to rats (Group B) had no significant effect on intestinal damage score compared to control animals (Group A) (Table 1). MTX-induced intestinal damage was manifested histologically by distortion of crypt architecture and degenerative changes of crypts such as: superficial epithelial atrophy, shortening of the villi, edema and polymorphonuclear leukocyte infiltration in the lamina propria. MTX-treated animals (Group C) demonstrated a significant increase in intestinal injury score in jejunum (two-fold increase, *p* = 0.005) and ileum (two-fold increase, *p* = 0.005) compared to control rats (Group A). Rats treated with MTX and enteral n-3 PUFA (Group D) as opposed to group C (MTX only) revealed a significant lower intestinal damage score in ileum (32%, *p* < 0.05) and a trend toward lower injury scores in jejunum; this trend did not reach statistical significance. CONTR-n-3 PUFA rats (Group B) exhibited a small but statistically significant fall in crypt depth in comparison with control (Group A) (Table 1). MTX-rats (Group C) showed a decrease in jejunal (17% decrease, *p* < 0.05) and ileum (18% decrease, *p* < 0.05) villus height, as well as a decrease in crypt depth in jejunum (11% decrease, *p* < 0.05) and ileum (9% decrease, *p* < 0.05) compared to control (Group A). Treatment of MTX rats with n-3 PUFA (Group D) resulted in a significant increase in villus height in ileum (17% increase, *p* < 0.05). Other results included increase in crypt depth in jejunum (12% increase, *p* < 0.05) and increased crypt depth in ileum (10% increase, *p* < 0.05) compared to Group C–MTX and no n-3 PUFA. 

#### 3.2.3. Pro-Inflammatory and Apoptosis Related Gene Expression (Real Time PCR)

**Figure 2:**
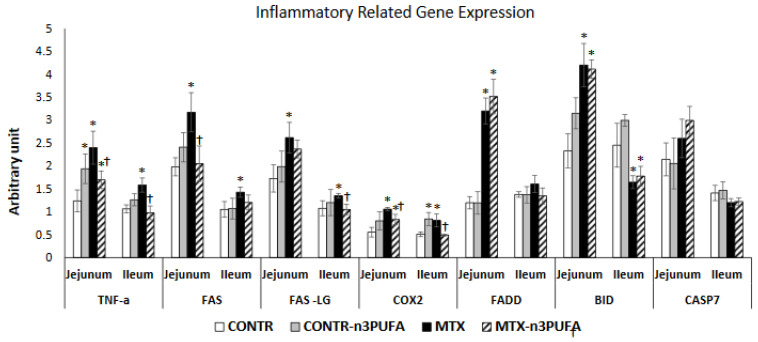
Effects of MTX and n-3 PUFAs on pro-inflammatory and apoptosis related gene expression. Real Time PCR was used to investigate gene expression. Relative changes in gene expression were normalized to housekeeping genes GAPDH and Tubulin. Values are mean ± SEM. CONTR-control, MTX-methotrexate, n-3 PUFAs- n-3 polyunsaturated fatty acids. * *p* < 0.05 all groups vs. CONTR rats, ^†^ *p* < 0.05 MTX-n-3 PUFAs vs. MTX rats.

Oral administration of n-3 PUFA in control rats (Group B) resulted in a small but significant increase in jejunal TNF-α (56%, *p* < 0.05) and BID (35%, *p* < 0.05) mRNA as well as ileal COX2 mRNA (65%, *p* < 0.05) expression compared to control animals (Group A) (Figure 2). MTX-animals (Group C) demonstrated a significant up-regulation in TNF-α (two-fold, *p* < 0.05), FAS (60%, *p* < 0.05), FasL (51%, *p* < 0.05), Fadd (2.5-fold increase, *p* < 0.05), Bid (two-fold increase, *p* < 0.05) and COX2 (two-fold increase, *p* < 0.05) mRNA in jejunum as well as in TNF-α (50%, *p* < 0.05), FAS (37%, *p* < 0.05), FasL (25%, *p* < 0.05), Fadd (17%, *p* < 0.05), and COX2(59%, *p* < 0.05) mRNA in ileum compared to control animals.MTX+n-3 PUFA rats (Group D) demonstrated a significant down-regulation in TNF-α mRNA in jejunum (29%, *p* < 0.05) and ileum (38%, *p* < 0.05), Fas mRNA in jejunum (35%, *p* < 0.05) and ileum (15%, *p* < 0.05), FADD mRNA in jejunum (16%, *p* < 0.05) and ileum (22%, *p* < 0.05), FasL mRNA in ileum (22%, *p* < 0.05), and COX2 mRNA in jejunum (22%, *p* < 0.05) and ileum (39%, *p* < 0.05) compared to MTX animals (Group C).

#### 3.2.4. TNF-α Levels in Plasma and Intestinal Tissue

**Figure 3:**
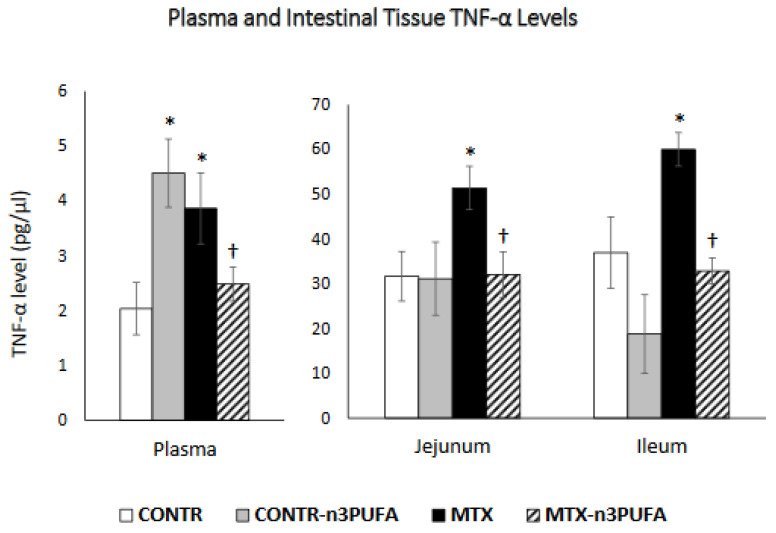
Changes in TNF-α levels in plasma and intestinal tissue during MTX induced intestinal damage and exposure to n-3 PUFAs. Enzyme-linked immunosorbent assay (ELISA) was used to determine TNF-α levels. Values are mean ± SEM. CONTR- control, MTX-methotrexate, n-3 PUFAs. * *p* < 0.05 all groups vs. CONTR rats, ^†^ *p* < 0.05 MTX-n-3 PUFAs vs. MTX rats.

CONTR+n-3 PUFA rats (Group B) demonstrated a significant increase (*p* = 005) in plasma TNF-α levels compared to control (Group A) (Figure 3). MTX-induced intestinal damage (Group C) resulted in a significant two-fold increase in TNF-α levels in plasma (*p* < 0.05) and a corresponding increase in TNF-α levels in jejunum (60% increase, *p* < 0.05) and ileum (62% increase, *p* < 0.05) compared to control. Oral administration of n-3 PUFA in MTX rats (Group D) resulted in a significant decrease in plasma (36%, *p* < 0.05) and intestinal tissue levels in jejunum (37%, *p* < 0.05) and ileum (45%, *p* < 0.05) in comparison with MTX-non-treated animals (Group C).

#### 3.2.5. Enterocyte Turnover (Proliferation and Apoptosis) and Intestinal Mucosal Inflammation Analysis

**Figure 4:**
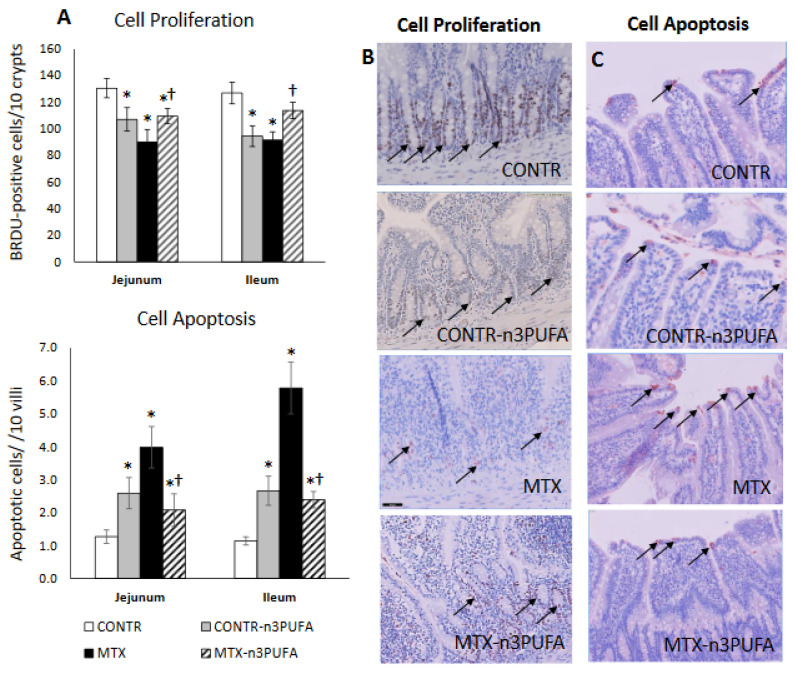
Effects of MTX and n-3 PUFAs on crypt cell proliferation (**A**,**B**) and apoptosis (**A**,**C**). Crypt cell proliferation was assessed using 5-bromodeoxyuridine (5-BrdU). Immunohistochemistry for Caspase-3 was performed for identification of apoptotic cells. Values are mean ± SEM. CONTR- control, MTX-methotrexate, n-3 PUFAs. * *p* < 0.05 all groups vs. CONTR rats, ^†^ *p* < 0.05 MTX+n-3 PUFAs vs. MTX rats.

Examining the proliferation and apoptosis in CONTR+n-3 PUFA rats (Group B), we noticed a decrease in enterocyte proliferation index in jejunum (107 ± 9 vs. 131 ± 7 BrdU positive cells/10 crypts, *p* < 0.05) and in ileum (95 ± 8 vs. 127 ± 8 BrdU positive cells/10 crypts, *p* = 0.01) and an accompanying two-fold increase in cell apoptosis both in jejunum (*p* < 0.05) and in ileum (*p* = 0.004) compared to controls (Group A) (Figure 4A). MTX induced damage was accompanied by disrupted architecture of intestinal crypts and progressive shift of the proliferative zone toward the crypt-villus junction (Figure 4B). MTX rats (Group C) demonstrated a decrease in enterocyte proliferation index in jejunum (91 ± 9 vs. 131 ± 7 BrdU positive cells/10 crypts, *p* = 0.004) and ileum (92 ± 6 vs. 127 ± 8 BrdU positive cells/10 crypts, *p* = 0.002) alongside a three-fold increase in apoptosis for jejunum (*p* < 0.001) and five–fold increase for ileum (*p* < 0.001) compared to controls (Group A).The proliferative zone of MTX+n-3 PUFA rats was only mildly affected, showing a slight upwards shift within the crypts. MTX+n-3 PUFA animals (Group D) showed an significant increase in cell proliferation in jejunum (21% increase, *p* < 0.05) and in ileum (23% increase, *p* < 0.05), While at the same time cell apoptosis fell by half in jejunum (*p* < 0.05) and in ileum (*p* < 0.001) compared to MTX-untreated rats. 

**Figure 5:**
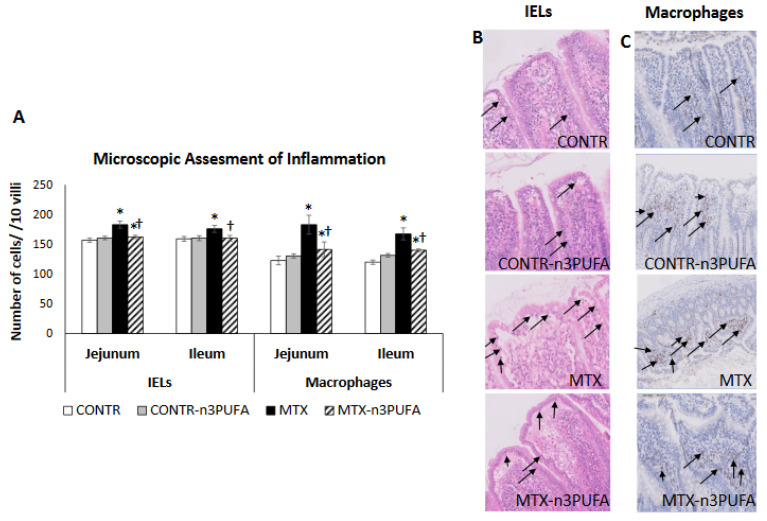
Grade of intestinal inflammation after administration of MTX and treatment with n-3 PUFAs. Immunohistochemistry with CD 68 antibody was performed to identify macrophages (**A**,**C**). The number of intraepithelial lymphocytes (IELs) was assessed by H&E staining and was calculated per 10 villi (**A**,**B**). Values are mean ± SEM. CONTR- control, MTX-methotrexate, n-3 PUFAs- n-3 polyunsaturated fatty acids. * *p* < 0.05 all groups vs. CONTR rats, ^†^ *p* < 0.05 MTX+n-3 PUFAs vs. MTX rats.

MTX-induced intestinal mucositis was accompanied by a 50% increase in macrophages presence in the jejunum (*p* < 0.05) and 40% increase in ileum (*p* < 0.05), as well as a rise in the number of intraepithelial lymphocytes (IELs) in jejunum (14% increase, *p* = 0.01) and ileum (11% increase, *p* = 0.01) in comparison with controls (Group A) (Figure 5). The decrease in the intestinal pro-inflammatory cytokine expression in MTX-n3PUFA rats (Group D) correlated with a decreased number of IELs in jejunum (11% decrease, *p* = 0.004) and ileum (10% decrease, *p* < 0.05) added to a decrease in the number of macrophages in jejunum (22% decrease, *p* < 0.05) and ileum (17% decrease, *p* < 0.05) compared to MTX-untreated group (Group C).

#### 3.2.6. Western Blot

**Figure 6:**
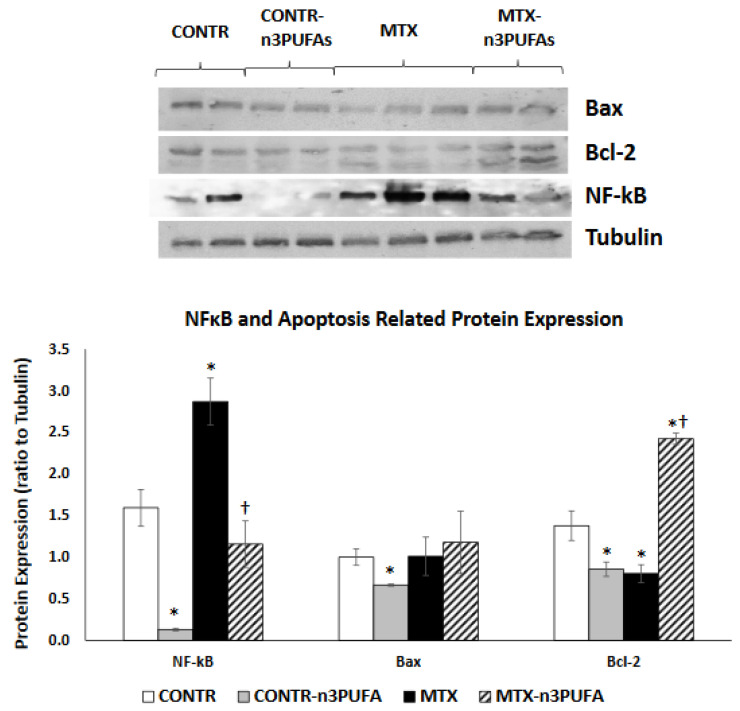
Changes in intestinal mucosal NF-κB, Bax and Bcl-2 protein levels following methotrexate induced intestinal damage and treatment with n-3 PUFAs. Values are mean ± SEM. CONTR-control, MTX-methotrexate, n-3 PUFAs. * *p* < 0.05 all groups vs. CONTR rats, ^†^ *p* < 0.05 MTX-n-3 PUFAs vs. MTX rats.

Rats treated with n-3 PUFA (Group B) demonstrated a significant decrease in NF-kB (10 fold decrease, *p* < 0.001), Bax (34% decrease, *p* = 0.003) and Bcl-2 (40% decrease, *p* < 0.05) protein expression in comparison with controls (Group A) (Figure 6). MTX-induced intestinal damage (Group C) was accompanied by a significant two-fold increase in NF-kB (*p* = 0.01) protein as well as in a concomitant 43% decrease (*p* < 0.05) in Bcl-2 protein levels. This finding correlated with the group’s increased rates of cell apoptosis. MTX+n-3 PUFA rats (Group D) showed a two-fold decrease in NF-kB (*p* = 0.003) protein as well as a concomitant three-fold increase (*p* < 0.001) in Bcl-2 protein levels which once again was in accordance with diminished cell apoptosis in this experimental group.

## 4. Discussion

Our current study was designed to investigate whether enteral supplementation with anti-inflammatory omega-3 fatty acids could reduce MTX-induced intestinal mucositis by reduction in levels of inflammatory cytokines in intestinal mucosa and serum and by decreasing intestinal epithelial-programmed cell death. The Exact mechanisms mediated by n-3 PUFA- which interfere with various inflammatory processes remain elusive. Their beneficial anti-inflammatory properties are reported to be a consequence of abundant accumulation in the sn-2 position of membrane phospholipids with cellular membrane conversion to an Omega-6/Omega-3 ratio close to 1:1, This process, which regulates membrane fluidity, causes a shift from inflammatory cytokines production, caused by scarcity of substrate to anti-inflammatory mediators. [18]. The ability of dietary n-3 PUFAs to decreases the production of various pro-inflammatory mediators such as IL-1, IL-2, IL-6 and TNF-α has be reported by many investigators [19]. In addition, n-3 PUFAs are able to decrease T-cell receptor expression, to reduce lymphocytes antigenic stimulation responses., to inhibit delayed-type hypersensitivity responses and IgA expression [20]. The positive anti-inflammatory effect of n-3 PUFAs has been described in several gastrointestinal inflammatory conditions. The use of Omega-3 FAs has been studied in the setting of patients suffering from inflammatory bowel disease (IBD) providing clinical evidence of decreasing disease activity, maintaining remission and in general improving patient’s quality of life. In the cell level the same study showed a decrease in bowel inflammation that might be attributed to a decrease noted of proinflammatory cytokines. [21]. The main mechanisms by which n-3 PUFAs may influence the development or course of IBD include inhibition of oxidative stress, TNF-α and proinflammatory cytokines production and decrease in expression of adhesion molecules [22]. 

Since reactive oxygen species release, DNA damage and DNA strands breaks production, release of endogenous damage-associated pattern molecules, activation of NF-κB cascade, encourage the generation of pro-inflammatory cytokines (TNF-α; IL-6; IL-1β), c-JUN and metalloproteinases (MMPs) are the critical events leading to chemotherapy-induced intestinal mucositis [23], we hypothesized in this study that n-3 PUFAs might play a part in reducing mucosal damage by suppressing the NF-κB/COX2 pathway with subsequent production of pro-inflammatory cytokines and by inhibiting cell death. 

The in-vitro part of the current study demonstrated a strong decrease in cell viability for both HT 29 and HCT 116 cell lines after being treated with MTX in comparison to control cells treated with vehicle alone. These data correlate with our previous experiment, which demonstrated that treatment with MTX of Caco-2 cells dramatically inhibited early cell proliferation and resulted in marked elevated levels of cell apoptosis, both in the early and late phase (compared to Caco-2 untreated cells) [24]. (vs exposure of non-treated with MTX cells to n-3 PUFA resulted in a decreased cell viability that was dose-independed for HT 29 cells and was concentration depended for HCT 166 cells. Similar results have been reported by other investigators. Several experiments have reported that n-3 PUFAs inhibit proliferation and stimulates cell apoptosis in different human colorectal adenocarcinoma cell lines, (HT-29, LS174T, and CO112) [25]. It is believed that n-3 PUFA incorporates into membrane phospholipids of highly proliferating cells, initiating free-radical chain reactions which lead to interaction of lipid peroxidation products with DNA, resulting in cell cycle arrest and apoptosis induction. The effect of increasing concentrations of n-3 PUFA on cell viability of MTX-treated cells was different from that of control cells treated with vehicle alone. Exposure of these cells to n-3 PUFA attenuated inhibiting effects of MTX on cell viability. MTX-n-3 PUFA cells showed an increased cell viability that was concentration non-depended for HT 29 cells and concentration depended for HCT 116 cells. These results are in contradiction to other in vitro studies, which have demonstrated that by inducing apoptosis through modulation of the caspase-8 or NF-κB pathway n-3 PUFAs actually amplifies the activity of anti-tumoural agents (such as TRAIL or docetaxel) [26].

We demonstrated in our animal experiment that treatment with n-3 PUFAs resulted in a mild inhibitory effect on epithelial cell turnover. This is evident from a small but significant decrease in crypt depth, decreased cell proliferation rate and increased cell apoptosis compared to control-nontreated animals. These findings are consistent with our in-vitro study (decreased cell viability may be correlated to increased cell apoptosis) as well as with several other clinical [27,28] and experimental [29] studies that have shown the importance of dietary n-3 PUFAs in the fine balance of cell turnover (apoptosis vs. proliferation) in colonic mucosa. 

Similar to our previous experiments [14,24], treatment of rats with MTX caused intestinal damage, apparent by higher scores of intestinal injury (compared to control animals), manifested by a loss of villus and crypt architecture, crypt remodeling, degeneration of some crypt and villi epithelial cells, villous epithelial atrophy, shortening of villi inflammatory mediators and mild hemorrhage in the lamina propria. Our experiment demonstrated changes in mucosal weight, villus height and crypt depth indicating that rats treated with MTX suffered from intestinal mucosal hypoplasia caused by decreased cell proliferation and increased cell apoptosis. The main regulator of small bowel cell loss during MTX-induced mucositis is apoptosis. Our results show that the both extrinsic and intrinsic pathways are responsible for stimulation of programmed cell death. The involvement of intrinsic pathways was evident from increased Bax and decreased Bcl-2 protein expression, consistent with changes in cell apoptosis. The critical role of Bcl-2 family and caspases in induction of crypt cell death during intestinal mucositis has been also described by other investigators [6]. Our study also shows that MTX-induced mucositis is accompanied by a significant increase in the pro-inflammatory cytokine TNF-α expression in plasma and remaining small intestinal mucosa. A significant up-regulation in TNF-α mRNA together with up-regulation of FAS, FasL, Fadd and Bid mRNA may suggest that the members of the extrinsic apoptotic pathway are also involved in regulation of MTX-induced intestinal epithelial cell apoptosis. MTX-treated rats also demonstrated a significant up-regulation of NF-κB (transcription factor nuclear factor kappa B) and COX-2 (cyclooxygenase-2) gene and protein levels. The role NF-κB induced pro-inflammatory cytokines and COX-2 production during development of mucositis has also previously described by other investigators [30]. NF-κB is known to play a part in modulating transcription of anti-apoptotic Bcl-2 family members [31]. 

A significant up-regulation in TNF-α expression in plasma and remaining small intestinal mucosa in our experiment was coupled with an increased number of macrophages and intestinal IELs, suggesting mucosal inflammation. IELs impart a crucial role in maintaining intestinal epithelial cell homeostasis and in modulation of intestinal immune response. Acute intestinal inflammation is usually accompanied by an increased number of IELs [32]. Abalo et al. demonstrated that high doses of 5-FU in rats significantly increased macrophage infiltration in small intestine (but not colon) [33]. 

Similar to our previous experiment findings [14], administration of enteral n-3 PUFAs in MTX rats protected the intestinal mucosa from MTX inflicted damage, evident by lower intestinal damage scores. Examining MTX+n-3 PUFAs rats’ intestines, we also noticed the presence of newly formed crypts and epithelial cell regeneration. These changes diminished inflammatory changes to lamina propria consequently decreasing atrophy. Treatment with n-3 PUFAs in our study resulted in a significant decrease in plasma and intestinal and TNF-α levels. This decrease in pro-inflammatory cytokine expression was coupled with a decrease in the number of macrophages and IELs in the remaining intestine suggesting diminished mucosal inflammation. Decreased production of NF-κB and COX-2 gene and protein levels may be responsible for decreased pro-inflammatory cytokine production by macrophages.n-3 PUFA exhibit anti-inflammatory properties in many inflammatory diseases including IBD, psoriasis, rheumatoid arthritis, chronic hepatitis etc. Omega-3 FAs reduce acute and chronic inflammatory reaction through various pathways. On one hand, n-3 PUFAs decrease activities of eicosanoids derived from arachidonic acid versus those derived from eicosapentaenoic acid. On the other hand, n-3 PUFAs is involved in the enhancement of anti-inflammatory lipid mediators such as resolvins and protectins. These suppress the activity of NF-kB with subsequently reducing production of enzymes and cytokines such as COX-2, TNF-α, and interleukin-1β that play a role in the inflammatory cascade [34]. In a recent study, Han et al. demonstrated that n-3 PUFAs prevent colitis-associated carcinogenesis through repressing NF-kB and inhibiting COX-2 production and by blocking dissociation of beta-catenin complex and inducing 15-prostaglandin dehydrogenase activity [35].

In addition to reduced inflammation, MTX+n-3 PUFA rats demonstrated enhanced intestinal repair. This was evident from increased overall bowel and mucosal weight as well as by increased villus height and crypt depth, which indicated that an increased intestinal volume can be attributed to cellular hyperplasia. Another explanation for increased cell mass in recovering bowel in rats fed withn n-3 PUFAs might also be activation of enterocyte turnover caused by enhanced cell proliferation and reduced apoptosis, compared to MTX-untreated rats. Inhibited cell apoptosis was accompanied in MTX+n-3 PUFA by increased Bcl-2 protein levels (together with unchanged Bax protein levels), but by a major decrease in TNF-α, Fas, FasL and FADD mRNA in mucosa, suggesting that extrinsic rather than intrinsic apoptotic pathway may be responsible for anti-apoptotic effects of n-3 PUFAs on intestinal mucosa.

In conclusion, dietary supplements with fish oil has proved to be an effective adjuvant therapy in colon cancer. In addition, n-3 PUFA might be of clinical importance in treating patients suffering from chemotherapy-induced oral and intestinal mucositis, preventing intestinal damage and stimulating intestinal recovery. Diminished NF-kB/COX-2 induced production of pro-inflammatory cytokines and inhibited cell apoptosis (mainly by inhibited extrinsic pathway) may be responsible for these positive effects. 

## Figures and Tables

**Table 1:** Effect of methotrexate and n-3 PUFAs on body weight gain, intestinal mucosal parameters and intestinal injury score.

Parameters	CONTR	CONTR+n-3 PUFA	MTX	MTX+n-3 PUFA
Body weight gain (%initial)	10.2 ± 1.9	8.2 ± 0.6	3.2 ± 1.6 *	6.5 ± 1.0 *^,†^
Bowel weight (mg/cm/100gBW) Jejunum Ileum	19.6 ± 1.2 19.2 ± 0.9	19.3 ± 0.5 18.4 ± 1.0	15.0 ± 0.9 * 15.5 ± 0.2 *	19.5 ± 1.1 ^†^ 18.1 ± 0.8 ^†^
Mucosal weight (mg/cm/100gBW) Jejunum Ileum	8.8 ± 0.9 7.7 ± 0.6	7.3 ± 0.2 7.1 ± 0.3	5.5 ± 0.5 * 5.6 ± 0.2 *	8.2 ± 0.8 ^†^ 7.7 ± 0.6 ^†^
Intestinal injury score Jejunum Ileum	1.6 ± 0.2 2.4 ± 0.4	1.6 ± 0.5 3.3 ± 0.4	3.0 ± 0.3 * 4.6 ± 0.7 *	2.3 ± 0.5 3.1 ± 0.4 ^†^
Villus height (µm) Jejunum Ileum	1492 ± 40 875 ± 45	1352 ± 85 882 ± 66	1241 ± 98 * 719 ± 42 *	1385 ± 72 842 ± 34 ^†^
Crypt depth (µm) Jejunum Ileum	214 ± 3 196 ± 4	209 ± 8 180 ± 7 *	191 ± 12 * 179 ± 10 *	214 ± 7 ^†^ 197 ± 5 ^†^

Values are mean ± SEM. CONTR- control, MTX-methotrexate, n-3 PUFAs. * *p* < 0.05 all groups vs. CONTR rats, ^†^ *p* < 0.05 MTX-n-3 PUFAs vs. MTX rats.

## Data Availability

Data is secured on hard drive under password on hospital computer and may be accessed by Igor Sukhotnick and Tal Koppelmann only. No data has been publically released.
